# Brazilian oral herbal medication for osteoarthritis: a systematic review protocol

**DOI:** 10.1186/s13643-016-0261-1

**Published:** 2016-05-21

**Authors:** Mariana Del Grossi Moura, Luciane Cruz Lopes, Maique Weber Biavatti, Jason W. Busse, Li Wang, Sean Alexander Kennedy, Neera Bhatnaga, Cristiane de Cássia Bergamaschi

**Affiliations:** Department of Pharmaceutical Sciences, University of Sorocaba, Sorocaba, São Paulo Brazil; Pharmaceutical Department, Federal University of Florianopolis, Florianopolis, Santa Catarina Brazil; Department of Clinical Epidemiology and Biostatistics, McMaster University, Hamilton, Ontario Canada; Department of Anesthesia, Michael G. DeGroote Institute for Pain Research and Care, McMaster University, Hamilton, Ontario Canada; Department of Anesthesia, McMaster University, Hamilton, Ontario Canada; Department of Diagnostic Radiology, University of Toronto, Toronto, Ontario Canada; Health Sciences Library, McMaster University, Hamilton, Ontario Canada

## Abstract

**Background:**

Osteoarthritis affects 1 % of the world’s population and is the most common cause of musculoskeletal impairment in the elderly. Herbal medications are commonly used in Brazil to manage symptoms associated with osteoarthritis, and some of them are financed by the Brazilian government; however, the effectiveness of most of these agents is uncertain. The aim was to systematically review the efficacy and safety of 13 oral herbal medications used in Brazil for the treatment of osteoarthritis.

**Methods:**

Randomized clinical trials eligible for our systematic review will enroll adults with osteoarthritis treated by a Brazilian herbal medication or a control group (placebo or active control). Using terms to include all forms of osteoarthritis combined with herbal medications, we will search the following electronic databases: Cochrane Central Register of Controlled Trials (CENTRAL); MEDLINE; EMBASE; CINAHL; Web of Science; Health Star; AMED, the database of the Cochrane Complementary Medicine Field, LILACS; CAB abstracts, Clinical trial.gov, WHO trials registry, and Bank of Brazil Thesis (CAPES), to 31 January 2016, without restrictions concerning language or status of publication. Outcomes of interest include the following: symptom relief (e.g., pain), adverse events (gastrointestinal bleeding, epigastric pain, nausea, and allergic reactions), discontinuation due to adverse events, quality of life, and the satisfaction with the treatment. Dichotomous data will be summarized as risk ratios; continuous data will be given as standard average differences with 95 % confidence intervals. A team of reviewers will assess each citation independently for eligibility and in duplicate it. For eligible studies, the same reviewers will perform data extraction, bias risk assessment, and determination of the overall quality of evidence for each of the outcomes using the Grading of Recommendations Assessment, Development and Evaluation (GRADE) classification system.

**Discussion:**

This is the first study that will evaluate the use of herbal medications used in Brazil for the treatment of pain caused by osteoarthritis. The results could guide prescribers in decision-making in clinical practice, to inform the patients with pain caused by osteoarthritis in relation to effective and safe treatment options and to inform the managers of the public health system which of the plants could actually be financed by the Brazilian government.

**Systematic review registration:**

PROSPERO 42015019793

**Electronic supplementary material:**

The online version of this article (doi:10.1186/s13643-016-0261-1) contains supplementary material, which is available to authorized users.

## Background

Osteoarthritis is the most common musculoskeletal disease and is associated with significant functional decline and reduced quality of life [1]. It is characterized by loss of articular cartilage, subchondral bone remodeling, bone spurs, ligament laxity, weakening of the periarticular muscles, and thickening of the capsule and synovial membrane [2–4]. Osteoarthritis is the result of both mechanical and biological events that cause imbalance in the normal process of degradation and synthesis of joint cartilage chondrocyte, extracellular matrix, and subchondral bone [5].

The World Health Organization (WHO) states that osteoarthritis is a disease related to an aging population [6] and the leading cause of chronic disability in middle-aged and older populations [7]. The risk of osteoarthritis increases from 1 % in 30 years old people to almost 10 % in people over the age of 40 years and 50 % in people over the age of 60 years [8]. Osteoarthritis produces a variety of serious social problems, both health and economic and is one of the more debilitating musculoskeletal diseases among the elderly [9].

Osteoarthritis can be associated with pain, stiffness, and functional limitations [10–12]. It is estimated to affect 10 % of men and 18 % of women and occurs most often in the hip and knee [13].

Although treatment guidelines recommend analgesics as first-line drugs, the non-steroidal anti-inflammatory drugs are preferred, although they are less safe and more expensive [14]. Due to the high incidence of adverse events related to non-steroidal anti-inflammatory drugs (NSAID) and the high costs associated with adverse events (e.g., gastrointestinal bleeding or perforation, additional medical visits, diagnostic procedures, treatments, and hospitalizations), therapeutic alternatives are an area of great interest [15, 16].

The use of herbal medicines worldwide is substantial and increasing. In 2001, the USA, around 38 % of adults and 12 % of children report use of herbal medicine [17]. Use of herbal medicines in developing countries is even greater, and an estimated 85 % of the Brazilian population use plants or preparations of these for their healthcare [18]. In 2011, the Brazilian herbal market generated 1.1 billion in revenue, which included sales of 43 million units of phytomedicines [19].

In primary health care, the use of medicinal plants has been stimulated by guidelines from various national health conferences and by the WHO [20]. The National Policy of Integrative and Complementary Practices and the National Policy of Medicinal and Phytotherapeutic Plants adopted in 2006 were created to meet the demands of the Brazilian population. These policies were decisive steps towards introducing the use of medicinal and phytotherapeutic plants in the Brazilian Unified Health System (SUS) [21].

In Brazil, there are 13 herbal medications marketed for the treatment of osteoarthritis: *Harpagophytum procumbens* DC. ex Meisn., *Uncaria tomentosa (Willd*.) DC., *Salix alba* L., (financed by the government), *Boswellia serrata* Roxb. ex Colebr., *Bowdichia virgilioides* Kunth., *Curcuma longa L*. (or *Curcuma domestica* Valeton), *Chenopodium ambrosioides* L., *Cordia curassavica (*Jacq.) Roem. & Schult. (or *Cordia verbenacea* DC.), *Salix daphnoides Vill*, *Salix purpurea L*., *Persea* gratissima Gaertn.f. (or *Persea americana* Mill.), *Uncaria guianesis* (Aubl.) J.F. Gmel, and *Zingiber officinale* Roscoe.

Two systematic reviews evaluated the use of herbal medicines for the treatment of osteoarthritis by topical and oral use, respectively [22, 23]. However, these studies did not include some of the plants marketed in Brazil: *B. virgilioides* Kunth, *C. ambrosioides* L, *C. curassavica* (Jacq.) Roem. & Schult, *S. alba* L., and *U. tomentosa* (Willd.) DC. Of these plants, *U. tomentosa* (Willd.) DC. and *S. alba* L. are funded by the Brazilian government to use in the Unified Health System (SUS), and *C. ambrosioides* L. and *C. curassavica* (Jacq.) Roem. & Schult are part of a list of plants of interest for development of research in order to include them as medicines financed by SUS.

Despite the common use of herbal medicines for managing osteoarthritis in adults, the safety and efficacy of some of these agents are uncertain. We therefore will conduct a systematic review of randomized controlled trials, which made use of oral herbal medicines used in Brazil for the treatment of osteoarthritis.

## Methods

### Standards

The systematic review will be performed according to the recommendations specified in the Cochrane Handbook for Interventional Reviews and reported according to the Preferred Reporting Items for Systematic Reviews and Meta-Analyses (PRISMA) statement [24] (see Additional file 1).

### Protocol and registration

We registered our review protocol in the International Prospective Register of Systematic Reviews (PROSPERO-CRD42015019793—http://www.crd.york.ac.uk/PROSPERO/).

### Eligibility criteria

#### Inclusion criteria

Patients: Adults (>18 years old) with a diagnosis of osteoarthritis according to the criteria of American College of Rheumatology (ACR): Western Ontario and McMaster Universities (WOMAC) [25] or the equivalent criterion of European League Against Rheumatism (EULAR): Lequesne index [26].

Interventions: One of the 13 oral herbal medicines is used by the Brazilian population from any of the following plant preparations (whole, powder, extract, crude drug, standardized mixture, and drug extract ratio and solvent): *B. serrata* Roxb. ex Colebr., *B. virgilioides* Kunth., *C. longa* L. (or *C. domestica* Valeton), *C. ambrosioides* L., *C. curassavica (*Jacq.) Roem. & Schult. (or *C. verbenacea* DC.), *H. procumbens* DC. ex Meisn., *Persea* gratissima Gaertn.f. (or *P. americana* Mill.), *S. alba* L., *S. daphnoides* Vill, *S. purpurea* L., *U. tomentosa* (Willd.) DC., *U. guianesis* (Aubl.) J.F. Gmel, and *Z. officinale* Roscoe. We will identify the daily dose, the active principles, and the marker substance of each plant. We will also investigate if each herbal medicine was prepared according to the WHO recommendations for the manufacturing procedure of medicinal plant parts (http://apps.who.int/medicinedocs/en/d/Jh2984e/).

Type of study: Randomized controlled trials including a group in which patients received one of the herbal medications listed above compared to a control group in which patients receive placebo or a non-herbal medicine controls (for example, NSAID).

#### Exclusion criteria

Patients: Studies in which more than 20 % of patients have other associated disease.

Interventions: Studies that investigated the simultaneous use of more than one of the eligible plants will be excluded.

### Measure outcomes

Our outcomes will be consistent with those proposed by the Cochrane musculoskeletal group systematic intervention reviews for osteoarthritis [27]. When necessary, the results will be evaluated to unification of the different scales.

Primary outcomes:

- Pain in overall or on walking (visual analogue scale (VAS), pain scale sub WOMAC; and other scales)
- Physical function—global disability or walking disability (sub-function range of WOMAC index and other scales)
- Swelling (VAS and other scales)
- Stiffness (WOMAC index and other scales)
- Quality of life (Short Form-36 and other scales)

Secondary outcomes:

- Adverse events: withdrawals and serious adverse events (that cause death, life-threatening, hospitalization, disability or permanent damage)
- Number of patients reporting any adverse effects
- Activity limitations
- Satisfaction with the treatment
- Consume of rescue medication
- Duration of symptom resolved
- Change in the structure of the joint (according to American College of Rheumatology criteria for osteoarthritis classification)

## Search methods for primary studies

### Electronic searches

We will search the following electronic databases without language restrictions: the Cochrane Central Register of Controlled Trials (CENTRAL) part of The Cochrane Library, MEDLINE, EMBASE, Cumulative Index to Nursing and Allied Health Literature (CINAHL), Web of Science, Health Star (via OVID), AMED, LILACS, CAB abstracts, clinical trial.gov, the WHO Trial Register and the Brazilian thesis database (CAPES), and trial register in Brazil (REBEC) to 31 January 2016; without language and status of publication restrictions. We will combine terms that describe osteoarthritis and herbal medications, individually.

### Searching other resources

We will review the reference list of every eligible study we identify and relevant review articles for additional eligible trials. We will write to the authors of all eligible trials and the pharmaceutical companies involved in the production of herbal medicines and inquire about additional trials of which they are aware of. Five Brazilian scientific journals will also be searched by hand for additional eligible studies (*Journal of Basic and Applied Pharmaceutical Sciences*, *Brazilian Journal of Pharmacy*, *Brazilian Journal of Pharmacognosy*, *Brazilian Journal of Medicinal Plants*, and *Brazilian Journal of Pharmaceutical Sciences*). Unpublished studies will be identified by searching in reference lists reported in the Brazilian legislation and conference proceedings (Medicinal Symposium of Brazilian medicinal plants; International Congress of Ethnopharmacology).

### Search strategy

The search will be conducted individually for each plant. We will use the following MeSH terms: (1) intervention (scientific name of plant, synonymies of each medicinal plant; popular name of each medicinal plant); (2) condition (osteoarthritis, ostepathritis, osteoarthritides, osteoarthrosis, osteoarthroses, arthritis, degenerative, arthritides, degenerative, degenerative arthritides, degenerative arthritis, and osteoarthrosis deformans). We will adapt the search strategy for each database. MEDLINE search strategy is provided in Table 1.

**Table 1:** Search strategy for *Harpagophytum procumbens* DC. ex Meisn. by MEDLINE (Via Ovid)

o #1 exp Osteoarthritis/or Osteoarthrits.mp. (57958) o #2 Osteoarthritides.mp. or exp Osteoarthritis/(44712) o #3 Osteoarthrosis.mp. or exp Osteoarthritis/(45663) o #4 Osteoarthroses.mp. or exp Osteoarthritis/(44719) o #5 Arthritis, Degenerative.mp. or exp Osteoarthritis/(44737) o #6 Arthritides, Degenerative.mp. or exp Osteoarthritis/(44711) o #7 Degenerative Arthritides.mp. or Osteoarthritis/(44718) o #8 Degenerative Arthritis.mp. or exp Osteoarthritis/(45350) o #9 Osteoarthrosis Deformans.mp. or exp Osteoarthritis/(44727 o #10 1 or 2 or 3 or 4 or 5 or 6 or 7 or 8 or 9 (59473) o #11 Harpagophytum.mp. or exp Harpagophytum/(149) o #12 Harpagophytums.mp. or exp Harpagophytum/(83) o #13 Harpagophytum procumbens.mp. or exp Harpagophytum/(135) o #14 Harpagophytum procumben.mp. or exp Harpagophytum/(82) o #15 procumben, Harpagophytum.mp. or exp Harpagophytum/(82) o #16 procumbens, Harpagophytum.mp. or exp Harpagophytum/(82) o #17 Devils Claw.mp. or exp Harpagophytum/(116) o #18 Claw, Devils.mp. or exp Harpagophytum/(83) o #19 Claws, Devils.mp. or exp Harpagophytum/(82) o #20 Devils Claws.mp. or exp Harpagophytum/(82) o #21 Exp Harpagophytum/(82) o #22 Exp Harpagophytum/(82) o #23 Harpagophytum/(82) o #24 11 or 12 or 13 or 14 or 15 or 16 or 17 or 18 or 19 or 20 or 21 or 22 or 23 (164) o #25 10 and 24 (37)	

### Eligibility determination

Four reviewers (CC, MG, MB, and SK), working in pairs, will independently screen potentially relevant citations and abstracts and will apply the selection criteria. We will obtain full texts of all articles that either reviewer feels might be eligible. Two reviewers will independently assess the eligibility of each full-text article and resolve disagreements by consensus. In case of duplicate publication, we will use the article with the more complete data.

### Data extraction

Four reviewers (CC, MG, MB, and SK), working in pairs, will independently extract the data and will record information regarding patients, methods, interventions, outcomes, and missing outcome data using standardized and pretested data extraction forms with instructions. Before starting data abstraction, we will conduct calibration exercises to ensure consistency between reviewers. We will contact study authors to resolve any uncertainties. Disagreements will be resolved by consensus with any unresolved issues referred to another reviewer.

### Risk of bias in individual studies

Using a modified version of the Cochrane collaboration risk of bias tool [28, 29], the same pairs of reviewers will independently asses the risk of bias for each randomized trial, according to the following criteria: random sequence; allocation concealment; blinding of the patient, healthcare professionals, outcome assessors, data collectors, and data analysts; incomplete outcome data; selective outcome reporting; and major baseline imbalance. Reviewers will assign response options of “definitely yes”, “probably yes”, “probably no”, and “definitely no” for each of the domains, with definitely yes and probably yes ultimately being assigned a low risk of bias and definitely no and probably no a high risk of bias [30]. Reviewers will resolve disagreements by discussion, and one arbitrator (LL) will adjudicate unresolved disagreements.

Possible explanations for heterogeneity will include the following: doses (higher versus lower) with an expected larger effect with higher doses, duration of the treatment (longer versus shorter) with an expected larger effect with longer duration of the treatment; and the risk of bias, with an expected larger effect in trials at high or unclear risk of bias versus trials at low risk of bias. We will assess heterogeneity associated with pooled effect estimates with the use of a *χ*^2^ test and the *I*^2^ statistic [31]. The following heterogeneity will be considered: 0 to 40 % (no important heterogeneity); 30 to 60 % (moderate heterogeneity); 50 to 90 % (substantial heterogeneity); and 75 to 100 % (considerable heterogeneity).

### Confidence in pooled estimates of effect

We will also independently rate the quality of evidence from randomized trials for each of the outcomes by using GRADE approach [32, 33]. In the GRADE approach, randomized trials begin as high-quality evidence but may be rated down by one or more of five categories of limitations: risk of bias, inconsistency, indirectness, imprecision, and reporting bias.

To measure agreement between the examiners, we will use the kappa statistics. Values of kappa between 0.40 and 0.59 have been considered to reflect fair agreement, values between 0.60 and 0.80 reflect good agreement, and values that are 0.75 or more reflect excellent agreement [34].

### Data synthesis

We will conduct analyses for each herbal intervention and pool of them for each outcome of interest. We will determine the confidence in estimates for each body of evidence and conduct an analysis for the body of evidence that warrants greater confidence. If the two bodies of evidence warrant similar confidence, we will conduct analyses for both bodies of evidence.

Meta-analyses will be conducted using Comprehensive Meta-Analysis STATA software (version 10.1). We will use random effects meta-analyses [35], which are conservative in that they consider within-studies and between-studies differences in calculating the error term used in the analysis. For trials that report dichotomous outcomes, we will calculate the pooled relative risk with associated 95 % confidence interval (95 % CI).

For continuous outcomes, e.g., pain score, function score, we will use weighted mean differences (WMD) and its 95 % CI as effect measure after we convert them into same scale of Western Ontario and McMaster Universities osteoarthritis index (WOMAC) pain score (0–100) and function score (0–100), in which high score indicates worse outcome. For quality of life, we will convert different scales to SF-36, in which high scores indicate better outcome. Once the WMD has been calculated, we will contextualize this value by noting, when available, the corresponding anchor-based minimally important difference (MID), the smallest change in instrument score that patients perceive is important.

If studies reported the same construct using different measurement instruments, we will calculate the standardized mean difference (SMD) as sensitivity analysis. The SMD expresses the intervention effect in standard deviation units, rather than the original units of measurement, with the value of an SMD depending on the size of the effect (the difference between means) and the standard deviation of the outcomes (the inherent variability among participants). For outcome measures that have an established anchor-based MID, we will use this measure to convert the SMD into an odds ratio and risk difference [36].

To facilitate the interpretation of the effects of continuous outcomes, we will substitute the MID, when MID is available for different scales, for the standard deviation (denominator) in the SMD equation, which will result in more readily interpretable MID units instead of standard deviation units [37]. If an estimate of the MID is not available, we will use a statistical approach developed by Suissa [36] to provide a summary estimate of the proportion of patients who benefit from treatment across all studies. Statistical approaches to enhance the interpretability of results of continuous outcomes outlined in this paragraph will use methods cited as well as those described by Thorlund et al. [38]. Funnel plots will be created to explore possible publication bias when at least 10 studies have contributed to a pooled analysis.

We will use recently developed approaches to address missing participant data for dichotomous outcomes [39] and continuous outcomes [40]. We will only apply these approaches to outcomes that meet the following criteria: show a significant treatment effect and report sufficient missing participant data to potentially introduce clinically important bias. Thresholds for important missing participant data will be determined on an outcome-by-outcome basis.

If sufficient studies are available, we will undertake subgroup analyses for doses (lower versus higher dose) and risk of bias (lower versus higher risk of bias). However, if the meta-analysis is not appropriate due to excessive heterogeneity of population, intervention, comparator, outcome, or methodology, we will construct summary tables and provide a narrative synthesis.

### Summarizing evidence

We will present results in evidence profiles as recommended by the GRADE working group [41, 42]. Evidence profiles provide succinct, easily digestible presentations of quality of evidence and magnitude of effects. Our evidence profiles will be constructed with the help of a software program, GRADEpro (http://ims.cochrane.org/gradepro) to include the following seven elements: (1) a list of until seven important outcomes, both desirable and undesirable; (2) a measure of the typical burden of these outcomes (e.g., control group, estimated risk); (3) a measure of the difference between risks with and without intervention; (4) the relative magnitude of effect; (5) numbers of participants and studies addressing these outcomes, as well as follow-up time; (6) a rating of the overall confidence in the estimate of effect for each outcome; and (7) comments, which will include the MID if available.

## Discussion

Our review will evaluate the available evidence for 13 oral Brazilian herbal interventions for osteoarthritis, provide estimates of the effectiveness of treatments and their associated harms, and evaluate the quality of the evidence in a thorough and consistent manner using the GRADE approach [43].

Previous systematic review had evaluated the oral use of herbal medicines to osteoarthritis [23]; however, five plants found in Brazilian market were not part of this review: *B. virgilioides* Kunth, *C. ambrosioides* L, *C. curassavica* (Jacq.) Roem. & Schult, *S. alba* L., and *U. tomentosa* (Willd.) DC; and the last two are financed by the Brazilian government. Despite the common use of oral herbal medications to manage osteoarthritis, these agents’ safety and effectiveness are uncertain.

We therefore will conduct a systematic review of these herbal medications used in Brazil for the treatment of osteoarthritis in order to guide prescribers in decision-making in clinical practice and to inform managers of the public health system which of these plants could actually be funded by the Brazilian government. The physician should opt for medication whose evidence is determined with the highest levels of quality in relation to effectiveness and safety. The results of our systematic review will be of interest for the public health system and practitioners worldwide, particularly in Brazil.

The compiled information about these herbal medications will inform patients and healthcare practitioners about their effectiveness and safety and help facilitate evidence-based shared care decision-making. The evidence of this study will allow health professionals to be aware of the effectiveness and safety of herbal medications used in Brazil for the treatment of osteoarthritis. This study will also identify key areas for future research.

## Additional file

Additional file 1: Preferred Reporting Items for Systematic Reviews and Meta-Analyses (PRISMA) statement. (DOCX 86.0 kb)

## Abbreviations

95 % CI: 95 % confidence interval
ACR: American College of Rheumatology
CAPES: Bank of Brazil Thesis
CENTRAL: Cochrane Central Register of Controlled Trials
CINAHL: Cumulative Index to Nursing and Allied Health Literature
EULAR: European League Against Rheumatism
GRADE: Grading of Recommendations Assessment, Development and Evaluation
MID: minimally important difference
NSAID: non-steroidal anti-inflammatory drugs
PRISMA: Preferred Reporting Items for Systematic Reviews and Meta-Analyses
SMD: standardized mean difference
SUS: Brazilian Unified Health System
VAS: visual analogue scale
WMD: weighted mean differences
WOMAC: Western Ontario and McMaster Universities
